# One-Step Room-Temperature Synthesis of Bimetallic Nanoscale Zero-Valent FeCo by Hydrazine Reduction: Effect of Metal Salts and Application in Contaminated Water Treatment

**DOI:** 10.1021/acsomega.2c03128

**Published:** 2022-09-20

**Authors:** Asmaa A. Koryam, Shaimaa T. El-Wakeel, Emad K. Radwan, Elham S. Darwish, Azza M. Abdel Fattah

**Affiliations:** † Water Pollution Research Department, National Research Centre, 33 El Buhouth St, Dokki, 12622 Giza, Egypt; ‡ Department of Chemistry, Faculty of Science, University of Cairo, 12613 Giza, Egypt

## Abstract

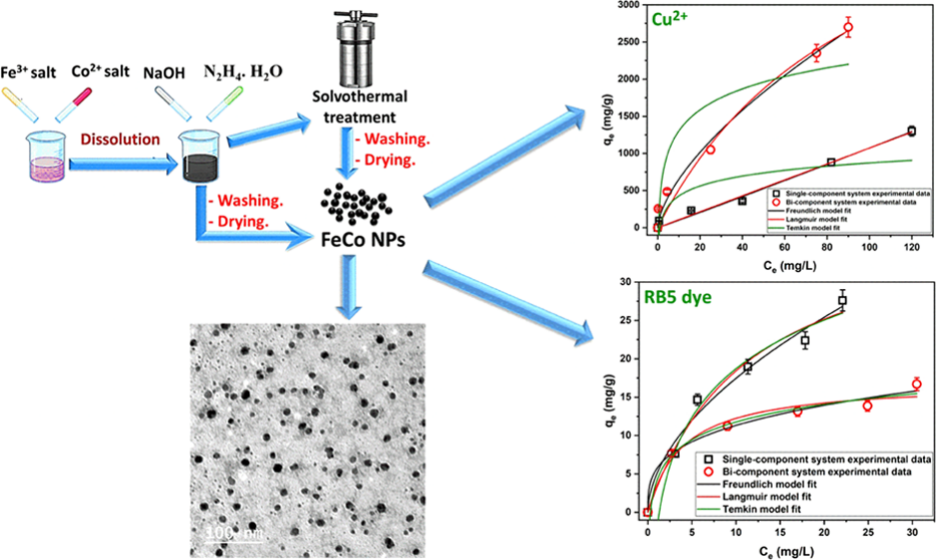

The effect of initial salt composition on the formation of zero-valent bimetallic FeCo was investigated in this work. Pure crystalline zero-valent FeCo nanoparticles (NPs) were obtained using either chloride or nitrate salts of both metals. Smaller NPs can be obtained using nitrate salts. Comparing the features of the FeCo prepared at room temperature and the solvothermal method revealed that both materials are almost identical. However, the room-temperature method is simpler, quicker, and saves energy. Energy-dispersive X-ray (EDX) analysis of the FeCo NPs prepared using nitrate salts at room temperature demonstrated the absence of oxygen and the presence and uniform distribution of Fe and Co within the structure with the atomic ratio very close to the initially planned one. The particles were sphere-like with a mean particle size of 7 nm, saturation magnetization of 173.32 emu/g, and surface area of 30 m^2^/g. The removal of Cu^2+^ and reactive blue 5 (RB5) by FeCo in a single-component system was conformed to the pseudo-first-order and pseudo-second-order models, respectively. The isotherm study confirmed the ability of FeCo for the simultaneous removal of Cu^2+^ and RB5 with more selectivity toward Cu^2+^. The RB5 has a synergistic effect on Cu^2+^ removal, while Cu^2+^ has an antagonistic effect on RB5 removal.

## 1 Introduction

Water quality is the main affair that occupies humankind as it is the source of life on Earth. Since our main goal is developing a sustainable ecological environment, reuse of wastewater is vital. The daily drainage of domestic and industrial wastewaters introduces different contaminants to the aquatic environment, which reduces water quality.^1^ Treatment of textile wastewaters is a worldwide concern since their release to the aquatic environment is fatal to aquatic and human life due to their complex nature that is composed of toxic trace metals and hazardous synthetic organic dyes.^2^ Among >10000 synthetic organic dyes, the reactive azo dyes are the most frequently used in the textile industry because of the simple dyeing procedure and covalent binding with cellulose fibers.^3,4^ These dyes cause an aesthetic problem, affect the photosynthesis process, form carcinogenic/mutagenic intermediates, and can harm the liver and kidney.^5,6^ On the other hand, knowledge about the harmful impact and the toxicity of trace metals has increased in the last two decades.^7^ Of the trace metals, copper is a very toxic abundant and naturally occurring element that has been detected in municipal wastewaters. It causes vomiting, cramps, convulsion, and even death if it enters the human body.^8^

Several treatment methods such as flocculation, ultrafiltration, and the biological method have been used in textile wastewater treatment and have been shown to be inefficient.^9−12^ On the contrary, the adsorption process, which is widely used, has been shown to be highly effective and more economical.^13,14^ Numerous materials have been used as adsorbents. Among them, magnetic zero-valent-based bimetallic nanoparticles (NPs) have attracted growing interest owing to their promising features. For instance, magnetic FeCo alloys are characterized by magnetic anisotropy energies, low magnetostriction, high resistivity, great coercivity, large saturation magnetization, obvious Snoek’s limit, and high Curie temperature.^15,16^ However, the preparation method determines the adsorption properties of FeCo alloys.

Both sodium borohydride and hydrazine hydrate are used as reducing agents in the preparation of zero-valent iron-based materials. However, hydrazine hydrate outperforms sodium borohydride because it produces high-purity Fe^0^ nanoparticles with good crystallinity and higher magnetization. From the environmental point of view, hydrazine hydrate produces harmless byproducts (nitrogen, hydrogen, and water) upon complete decomposition, and the waste stream of the hydrazine hydrate reduction process can be easily treated compared to that of sodium borohydride.^17−19^ Several methods based on hydrazine reduction have been reported to prepare the FeCo alloy such as the texture-controlled technique,^20^ the polyol process,^21^ hydrothermal synthesis,^22^ electrodeposition,^23^ mechanical alloying,^24^ reductive salt-matrix annealing,^25^ spray pyrolysis with hydrogen reduction,^26^ organometallic route,^27^ self-catalyzed coreduction method,^28^ ultrasonic wave-assisted solution method,^29^ solution phase method,^30^ and coprecipitation.^31^ However, some of these methods are complex, energy-intensive, and/or result in low-crystalline and impure FeCo alloys. In addition, the effect of the initial salt composition on the features of the formed FeCo has not been investigated yet. This work is devoted to filling this literature gap by studying, for the first time, the effect of initial salt composition on the features of the FeCo alloy. Furthermore, this work reports for the first time the simultaneous removal of anionic and cationic contaminants from contaminated water using FeCo alloys. More specifically, we report an investigation on the effect of iron and cobalt salts on the formation of nanosized zero-valent FeCo alloys using a simple one-step room-temperature quick method. In this method, the salts of iron and cobalt are reduced by hydrazine in an alkaline medium. In addition, the features of FeCo prepared by this method were compared to those prepared by the solvothermal method. The qualities of the prepared materials were investigated using X-ray diffraction (XRD), field emission scanning electron microscopy (FESEM), energy-dispersive X-ray (EDX), high-resolution transmission electron microscopy (HRTEM), a vibrating sample magnetometer (VSM), N_2_ adsorption at 77 K, and an X-ray photoelectron spectrometer (XPS).

The efficiency of the FeCo prepared at room temperature and solvothermal methods for the removal of reactive black 5 (RB5) dye and Cu^2+^ was compared. Finally, detailed parametric, kinetics, and isotherm studies were executed on the removal of RB5 and Cu^2+^ by the FeCo prepared at room temperature in single- and bicomponent systems.

## 2 Materials and Methods

Ferric nitrate nonahydrate, (Fe(NO_3_)_3_·9H_2_O), ferric chloride hexahydrate (FeCl_3_·6H_2_O), cobalt nitrate hexahydrate (Co(NO_3_)_2_·6H_2_O), cobalt chloride heptahydrate (CoCl_2_·6H_2_O), cobalt sulfate heptahydrate (CoSO_4_·7H_2_O), sodium hydroxide (NaOH), hydrazine hydrate (N_2_H_4_·7H_2_O), sodium chloride (NaCl), and copper sulfate pentahydrate (CuSO_4_·5H_2_O) were purchased from Sigma–Aldrich and used as received. Reactive blue 5 was obtained from a local dyeing factory.

### 2.1 Preparation Methods

#### 2.1.1 Room-Temperature Method

First, precise amounts of iron and cobalt salts were weighed to obtain a theoretical Fe^0^:Co^0^ mass ratio of 1:1 and then dissolved in 20 mL of ethanol. Then, 5.0 g of NaOH was added to the solution under stirring followed by 10 mL of hydrazine hydrate. Stirring was continued until homogeneity. The resulting magnetic product was collected by magnetic separation and washed with hot distilled water and then absolute ethanol several times. Finally, the products were dried in a vacuum oven at 40 °C. Table 1 gives the codes of the prepared samples and the salts used in their preparation.

**Table 1:** Salts Used in the Preparation of FeCo Alloy and the Code of Resulting Samples

sample code			
1	salt	Fe(NO_3_)_3_·9H_2_O	Co(NO_3_)_2_·6H_2_O
	weight	1.5390 g	0.9876 g
2	salt	Fe(NO_3_)_3_·9H_2_O	CoCl_2_·6H_2_O
	weight	1.5390 g	0.8075 g
3	salt	Fe(NO_3_)_3_·9H_2_O	CoSO_4_·7H_2_O
	weight	1.5390 g	0.9540 g
4	salt	FeCl_3_·6H_2_O	Co(NO_3_)_2_·6H_2_O
	weight	0.9680 g	0.9876 g
5	salt	FeCl_3_·6H_2_O	CoCl_2_·6H_2_O
	weight	0.9680 g	0.8075 g
6	salt	FeCl_3_·6H_2_O	CoSO_4_·7H_2_O
	weight	0.9680 g	0.9540 g

#### 2.1.2 Solvothermal Method

A typical solvothermal method using chloride salts of iron and cobalt (sample 5 in Table 1) was followed. The sample preparation followed the same steps as previously, but, after homogeneity, the mixture was transferred into a Teflon-lined stainless-steel autoclave and kept at 80 °C for 12 h. Afterward, the autoclave was left to cool down to room temperature, and the product was washed and separated as illustrated above. The resulting sample was coded as 7.

### 2.2 Characterization Methods

The crystal structure of the prepared materials was characterized by an X-ray diffractometer (Bruker AXS D8 advance instrument) between 5 and 80° (2θ). The morphology, composition, and metal distribution were obtained by JEOL 6400 F field emission scanning electron microscopy (FESEM) with energy-dispersive X-ray analysis (EDX). Transmission electron microscopy (JEOL TEM-2100) was used to characterize the internal structure of the materials. The magnetic properties were investigated through a vibrating sample magnetometer (LakeShore 7410). A BELSORP-max surface analyzer was used to obtain the N_2_ adsorption–desorption isotherms at 77 K. The Brunauer–Emmett–Teller specific surface area (*S*_BET_) and the nonlocal density functional theory (NLDFT) pore size distribution were determined from the obtained N_2_ adsorption–desorption isotherms. The surface elemental composition and states of the materials were investigated by an X-ray photoelectron spectrometer (XPS) (K-Alpha system, Thermo Fisher Scientific) with an X-ray Al K-Alpha monochromator source and an X-ray spot size of 50–400 μm. Full-spectrum range was acquired using a pass energy of 200 eV at a narrow spectrum of 50 eV.

The salt addition method was used to determine the pH of the point of zero charge (pH_pzc_) of the FeCo alloy.^32^ A series of sodium chloride solutions of concentration 0.01 mol/L was adjusted to initial pH (pH_o_) values of 2, 4, 6, 8, and 10 using 1 mol/L NaOH or HCl solutions. Afterward, 0.25 g of both FeCo alloys was added to 50 mL of each salt solution and shaken for 24 h at room temperature. The final pH was measured, and the changes in the solution pH values (ΔpH), calculated as the difference between the final and initial pHs, were plotted versus pH_o_. The pH_pzc_ was identified through the intersection of the curve with the *x*-axis (pH_o_).

### 2.3 Adsorption Study

First, the efficiency of the FeCo prepared by solvothermal and room-temperature methods toward Cu^2+^ and RB5 was compared. Then, the best sample was used to investigate the effects of the adsorbent dosage and initial pH (pH_o_) as a function of contact time on the adsorption process. The aforementioned experiments were performed in a single-component adsorption system. Finally, two types of adsorption isotherms were performed: isotherm with a single adsorptive (Cu^2+^ or RB5) and isotherm with binary mixtures of both Cu^2+^ and RB5. In the binary mixture, two sets of experiments were performed: one with a constant initial concentration of RB5 (30 mg/L) and a variable initial concentration of Cu^2+^ (10–250 mg/L), and the second with a constant initial concentration of Cu^2+^ (100 mg/L) and a variable initial concentration of RB5 (10–50 mg/L). All of the experiments were conducted in 250 mL conical flasks at room temperature using a speed-adjustable Orbital shaker. Samples were withdrawn at different time intervals; then, FeCo was magnetically separated and the remaining concentration of RB5 or Cu^2+^ was determined. A Jasco V730 was used to determine the concentration of the RB5 dye. Six different initial concentrations of the dye were measured to construct a calibration curve. The maximum wavelength was found at 598 nm. Figure S1 gives the UV–vis spectra of the RB5 dye and the calibration curve. Copper ion concentrations were determined using inductively coupled plasma optical emission spectrometry (ICP-OES, Agilent 5100) in the prepared samples. Details about the experimental conditions are given in the caption of figures, and details about the analysis of adsorption data, including kinetic and isotherm models and the criteria of selecting the best fitting model, are given in the Supporting Information.

## 3 Results and Discussion

### 3.1 Characteristics of the Prepared Materials

Hydrazine hydrate (N_2_H_4_·H_2_O) is a low-cost and low-impurity potent reducing agent; therefore, it has been widely used for the synthesis of several nanosized materials. In this work, the FeCo alloy was prepared by the reduction of Fe and Co salts using N_2_H_4_·H_2_O in an alkaline medium according to the following reactions
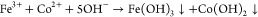
1
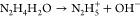
2
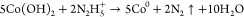
3
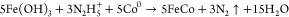
4

In these reactions, NaOH acts as a precipitant that converts the Fe and Co salts to their hydroxides (eq 1) and also enhances and maintains the reducing ability of N_2_H_4_·H_2_O (eq 2).

The formation of FeCo at ambient conditions starts by the reduction of Co^2+^ to Co^0^ (eq 3), which subsequently catalyzes the reduction of Fe^3+^ to Fe^0^ (eq 4). Thus, Co^0^ enters the lattice of Fe^0^ particles, forming the FeCo alloy.^30^ This sequence of reaction is due to the fact that the Co^2+^/Co^0^ reduction potential (−0.73 V) is higher than that of Fe^+3^/Fe^0^ (−0.77 V); therefore, N_2_H_4_·H_2_O can reduce Co^2+^ at ambient temperature and pressure, but the reduction of Fe^3+^ requires surface catalysis or high pressure.^29,30^ Noteworthy, the N_2_ generated during the reaction could protect the formed FeCo against oxidization.^33^

**Figure 1:**
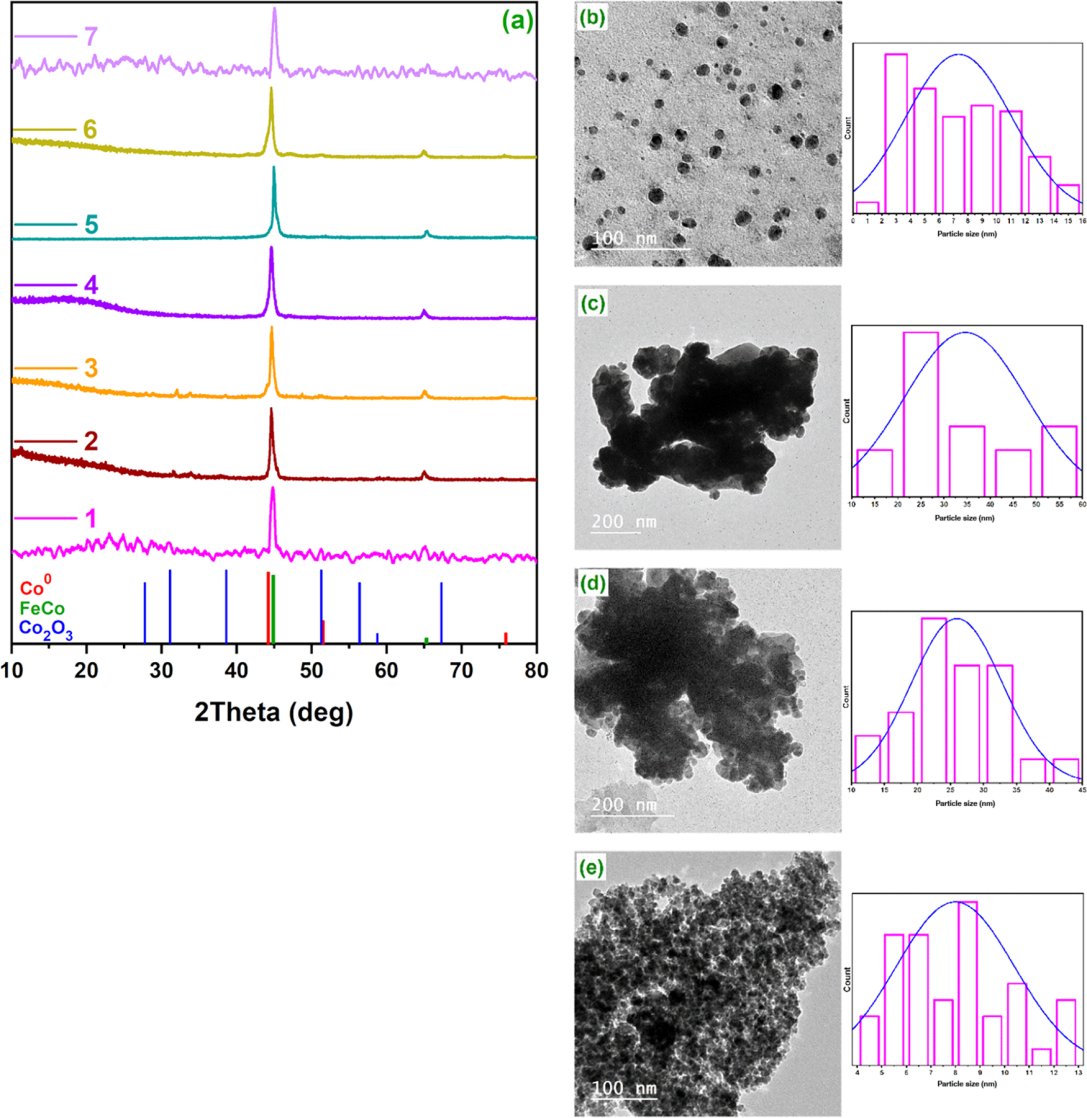
XRD (a) and HRTEM and particle size distribution histogram (b, c, d, and e) of FeCo sample nos. 1, 4, 5, and 7, respectively.

Figure 1a shows the XRD patterns of the samples prepared according to Table 1. All of the prepared samples show two diffraction peaks at 2θ = 44 and 65° characteristic of the crystal planes of (1 1 0) and (2 0 0) of the body-centered cubic (bcc) phase of the FeCo alloy (JCPDS card No: 49-1567). However, the purity of the samples is different. Relatively weak diffraction peaks could be observed in the patterns of sample no. 2 at 2θ = 31 and 38° and sample no. 3 at 2θ = 38 and 51°. These peaks indicate the presence of cobalt oxide (JCPDS card No: 02-0770), which implies a slight oxidation in these samples. In sample no. 6, diffraction peaks can be observed at 2θ = 51 and 75° and 2θ = 41 and 47°, which can be assigned to the face-centered cubic phase of Co^0^ (JCPDS card Nos: 15-0806 and 01-1277, respectively). Contrariwise, no impurity peaks can be noticed in samples no. 1, 4, 5, and 7, indicating the successful coreduction of Fe and Co salts and the formation of a highly crystalline pure single bcc-FeCo phase. Obviously, the intensity of the diffraction peaks of samples no. 1 and 7 is relatively low, which could be ascribed to the smaller particle size of these samples. To verify this assumption, the particle shape and size of these samples were determined by HRTEM and are displayed in Figure 1b–e.

Figure 1b–e reveals that sample nos. 1, 4, 5, and 7 are composed of sphere-like nanoparticles (NPs); however, the samples have different particle sizes and degrees of agglomeration. Sample nos. 4, 5, and 7 (Figure 1c–e) agglomerate into large clusters, while sample no. 1 is well dispersed. The aggregation of NPs creates several interparticle voids, which could result in high surface area.

It is known that the reactivity of Fe^0^-based bimetals increases as their particle size decreases.^34^ Therefore, the particle size of sample nos. 1, 4, 5, and 7 was compared to select the most reactive sample. The particle size of sample nos. 1 and 7 ranged between 2–16 and 4–13 nm with a mean of 7 and 8 nm, respectively. However, sample nos. 4 and 5 have particles with sizes ranging between 15–59 and 13–41 nm and a mean of 35 and 26 nm, respectively. Thus, it can be concluded that sample nos. 1 and 7 have similar particle sizes, which is significantly smaller than those of sample nos. 4 and 5.

Overall, the results of XRD and HRTEM indicate that the initial salt composition has a paramount effect on the purity and particle size of the produced FeCo. Although the anions of Fe and Co salts do not take part directly in the reactions of FeCo formation (eqs 1–3), they play a key role by constituting the ionic atmosphere, which affect the reaction rate. Increasing the ionic strength of the reaction solution causes a decrease in the reaction rate and favors the formation of smaller particles.^35^ Herein, the ionic strength of the solutions used for the preparation of sample no. 1 was calculated to be 3.30 mol/L, while that of sample nos. 4, 5, and 7 was 3.18 mol/L. Therefore, sample no. 1 has the smallest particle size and sample nos. 4 and 5 have comparable particle sizes. Subjecting sample no. 5 to a solvothermal treatment step to get sample no. 7 decreased the particle size considerably; the mean particle size decreased from 26 to 8 nm.

In a nutshell, the XRD and particle size results reflect that both solvothermal and room-temperature methods using Fe and Co nitrate salt methods can efficiently prepare pure highly crystalline nanoscale zero-valent bimetallic FeCo alloys with almost the same particle size. Therefore, the characteristics of both sample nos. 1 and 7 were further explored. Henceforward, sample nos. 1 and 7 were referred to as room-temperature and solvothermal methods, respectively.

**Figure 2:**
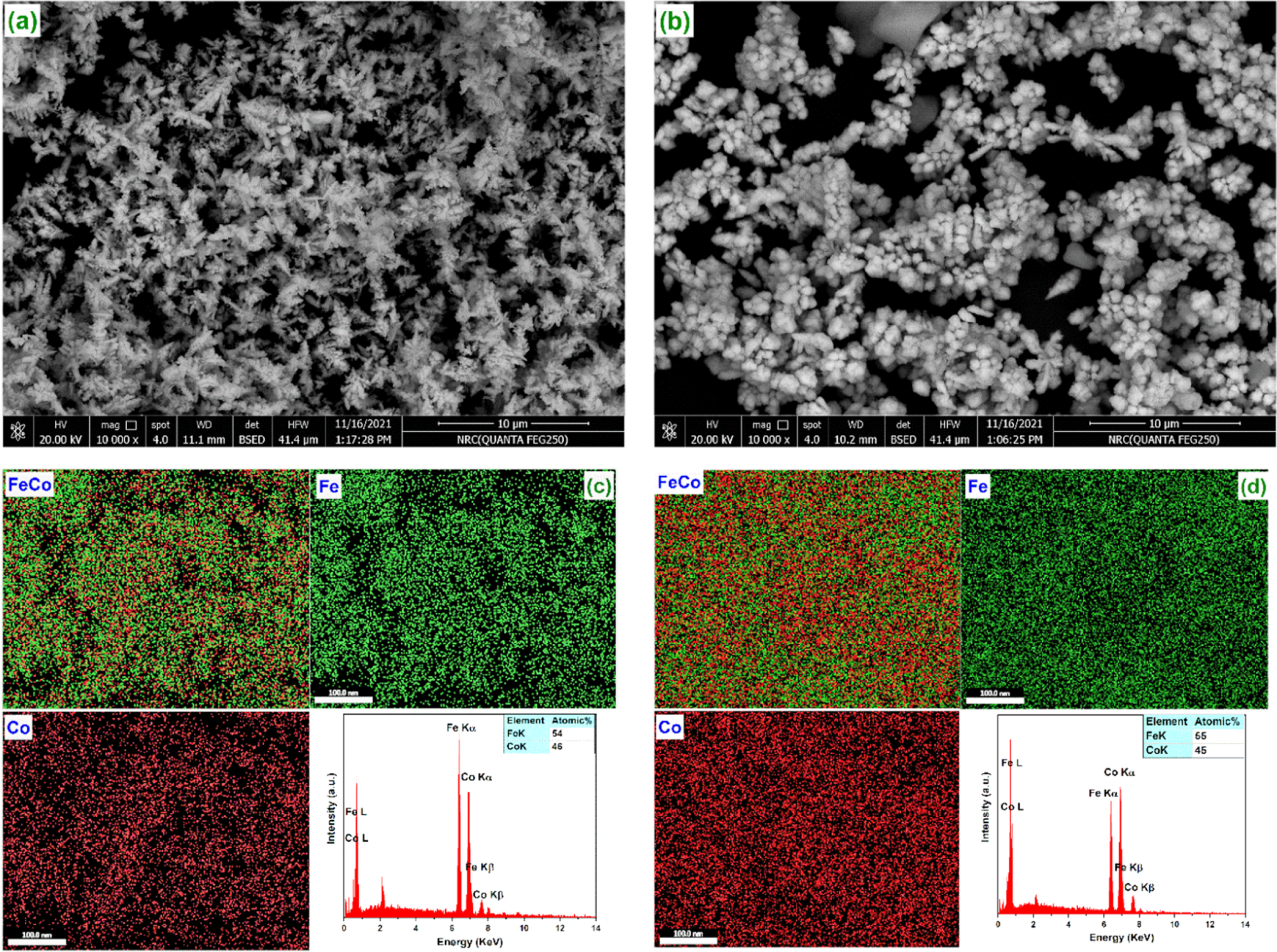
FESEM (a, b) and EDX and elemental mapping (c, d) of FeCo prepared by solvothermal and room-temperature methods, respectively.

Figure 2 compares the structure characteristics of FeCo prepared by solvothermal and room-temperature methods. The FESEM images (Figure 2a,b) show that FeCo produced by both methods forms large aggregates with interparticle voids. The attractive magnetic forces between the particles cause the formation of such aggregates.^36^ A higher degree of aggregation can be observed for the sample prepared by solvothermal methods (Figure 2a); consequently, this sample has more interparticle voids. Figure 2a shows that the sample prepared by the solvothermal method has a three-dimensional structure with spicate branches, which aggregate in an irregular shape. While the sample prepared at room temperature (Figure 2b) is mainly quasi-spherical with an irregular salient. Stacking of the initial particles results in the appearance of conelike and double-conelike structures as minor morphologies. The difference between the HRTEM and FESEM of the sample prepared at room temperature is due to the different pretreatment of the sample for each characterization technique.

The coexistence of Fe and Co elements, their atomic ratio, and their distribution were evaluated by EDX and EDX mappings. Figure 2c,d proves the presence of both Fe and Co in the samples prepared by both methods. Also, it can be noticed that the Fe and Co are uniformly distributed within the structure. The EDX spectra (Figure 2c,d) clarify that the presence of two peaks only corresponds to Fe and Co. Importantly, the EDX analysis shows the absence of an oxygen signal in the prepared samples, which further confirms the successful preparation of zero-valent FeCo. The atomic ratios of Fe/Co calculated from the EDX analysis were 54/46 and 55/45 for the sample prepared by solvothermal and room-temperature methods, respectively. Therefore, it can be concluded that both methods give almost the same elemental composition of the FeCo alloy. These ratios also indicate the closeness of the practical ratio to the initially designed composition.

**Figure 3:**
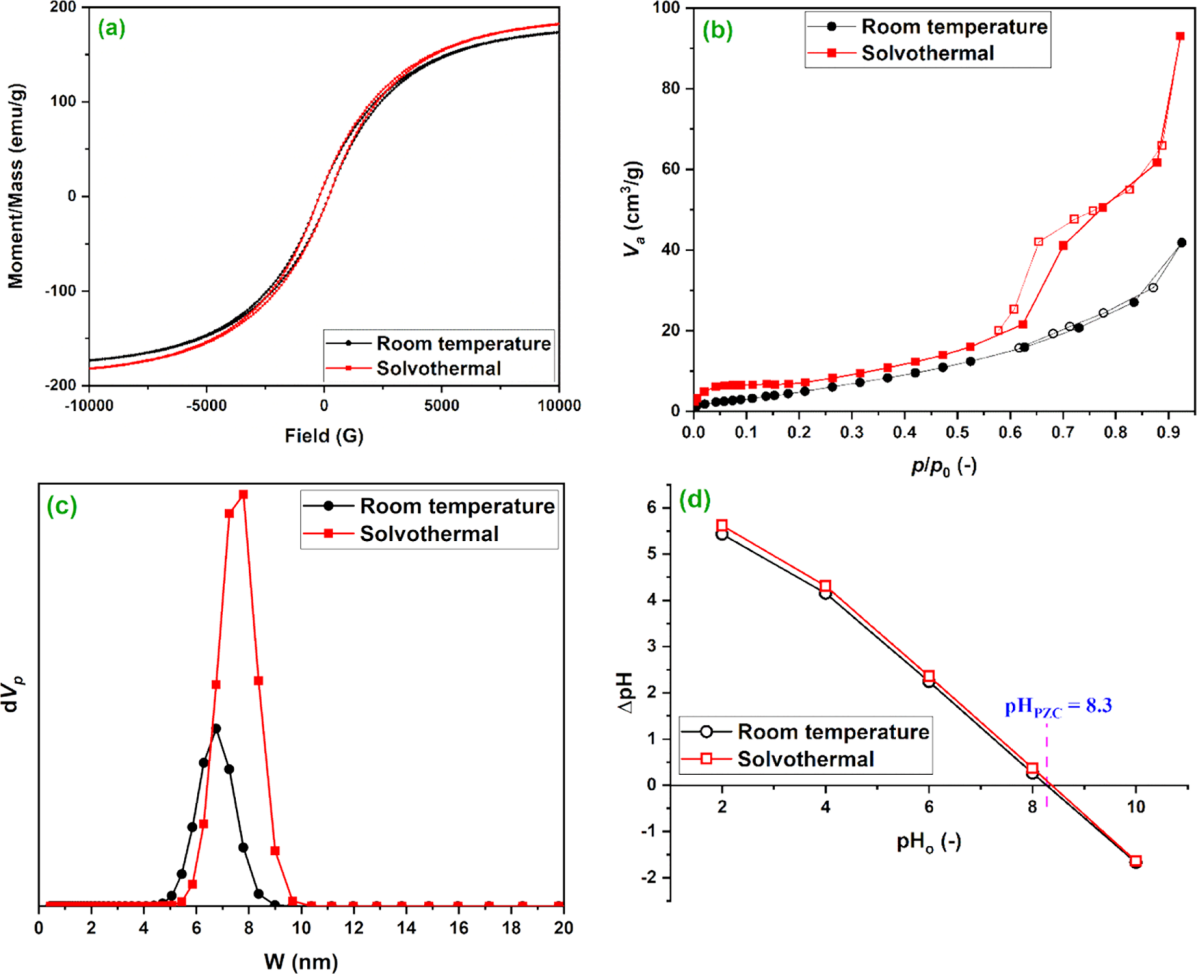
Magnetization curves (a), N_2_ adsorption–desorption isotherm (b), NLDFT pore size distribution curve (c), and pH_PZC_ (d) of bimetallic zero-valent FeCo nanoparticles prepared by solvothermal and room-temperature methods.

Figure 3a displays the room-temperature magnetization curves of the prepared samples, and Table 2 lists the values of magnetic parameters. The magnetization curves are S-shaped with a hysteresis loop, which is characteristic of ferromagnetic materials. The saturation magnetization of the sample prepared at room temperature (173.32 emu/g) is insignificantly lower than that of the sample prepared by the solvothermal method (182.02 emu/g). These values reflect the high capability of magnetic separation and the consequent high removal efficiency of the dispersed product from the aqueous medium, which encourages its application in water treatment. Table 2 also shows that both samples have almost the same coercivity and retentivity. Specifically, the coercivity values were 201.86 and 202.71 G for the samples prepared by solvothermal and room-temperature methods, respectively. However, the value of retentivity reached 12.92 emu/g for the sample prepared by the solvothermal method and 12.38 emu/g for the sample prepared at room temperature. Again, the results of the magnetic properties prove the close similarity between the two preparation methods.

**Table 2:** Magnetic and Textural Properties of FeCo NPs Prepared by Solvothermal and Room-Temperature Methods

	solvothermal method	room-temperature method
magnetization (emu/g)	182.08	173.32
coercivity (G)	201.86	202.71
retentivity (emu/g)	12.92	12.38
*S*_BET_ (m^2^/g)	32.59	29.76
pore size (nm)	7.25	6.75

The N_2_ adsorption–desorption isotherms of the two FeCo samples are shown in Figure 3b. The isotherms of both samples belong to the type II isotherm, while the hysteresis loops belong to type H3 of the IUPAC classification. The type II isotherm is typical for nonporous and macroporous materials, and the type H3 loop is common for nonrigid aggregates of particles and macroporous materials partially filled with pore condensate.^37^ Thus, the N_2_ adsorption–desorption isotherms suggest nonporous or macroporous materials. On the other hand, according to the NLDFT pore size distribution curve (Figure 3c and Table 2), mesopores dominate the porous structures of both samples, implying that both samples are mainly mesoporous. These results seem contradictory, but correlating the results of N_2_ adsorption, FESEM, and HRTEM could reveal the nature of the materials. The results of SEM and TEM illustrated that the materials are composed of aggregation of NPs. Thus, the suggested mesoporosity of the materials by NLDFT is a result of the interparticle voids of the aggregates, while the suggested nonporosity by the N_2_ adsorption–desorption can be attributed to the nature of FeCo NPs. In other words, the FeCo NPs are nonporous and their aggregation gives rise to mesopores. The specific surface area calculated by the multipoint Brunauer–Emmett–Teller method (*S*_BET_, Table 2) of the sample prepared by the solvothermal method (32.59 m^2^/g) was slightly higher than that of the sample prepared at room temperature (29.76 m^2^/g). This result agrees with the results of FESEM and HRTEM, which illustrated that the sample prepared by the solvothermal method has relatively larger aggregates with more interparticle voids than the sample prepared at room temperature.

The point of zero charge (pH_PZC_) pH_PZC_ is the pH value at which the material surface charge becomes neutral. It is of paramount importance for materials that are used as adsorbents as it gives an indication about the surface charges of the material at different pH values. Figure 3d displays the plot of ΔpH vs pH_o_. It can be observed that the pH_PZC_ of both samples is nearly identical and its value is 8.3. Thus, at pH > 8.3, the surface of FeCo NPs is negatively charged, whereas at pH < 8.3, the surface of FeCo NPs is positively charged.

Conclusively, the results of the characterization of the FeCo alloy prepared by both solvothermal and room-temperature methods using Fe and Co nitrate salts are almost identical; however, the preparation at room temperature is advantageous as it is simple and saves energy and time.

### 3.2 Contaminants’ Removal Properties

Zero-valent iron-based bimetallic nanoparticles can remove trace metals, oxyanions, and different types of organic contaminants. The mechanism of removal can be one or more of adsorption, coprecipitation, reduction, oxidation, hydrodehalogenation, hydrogenation, or hydrodeoxygenation based on the type of contaminant and experimental conditions.^38^

**Figure 4:**
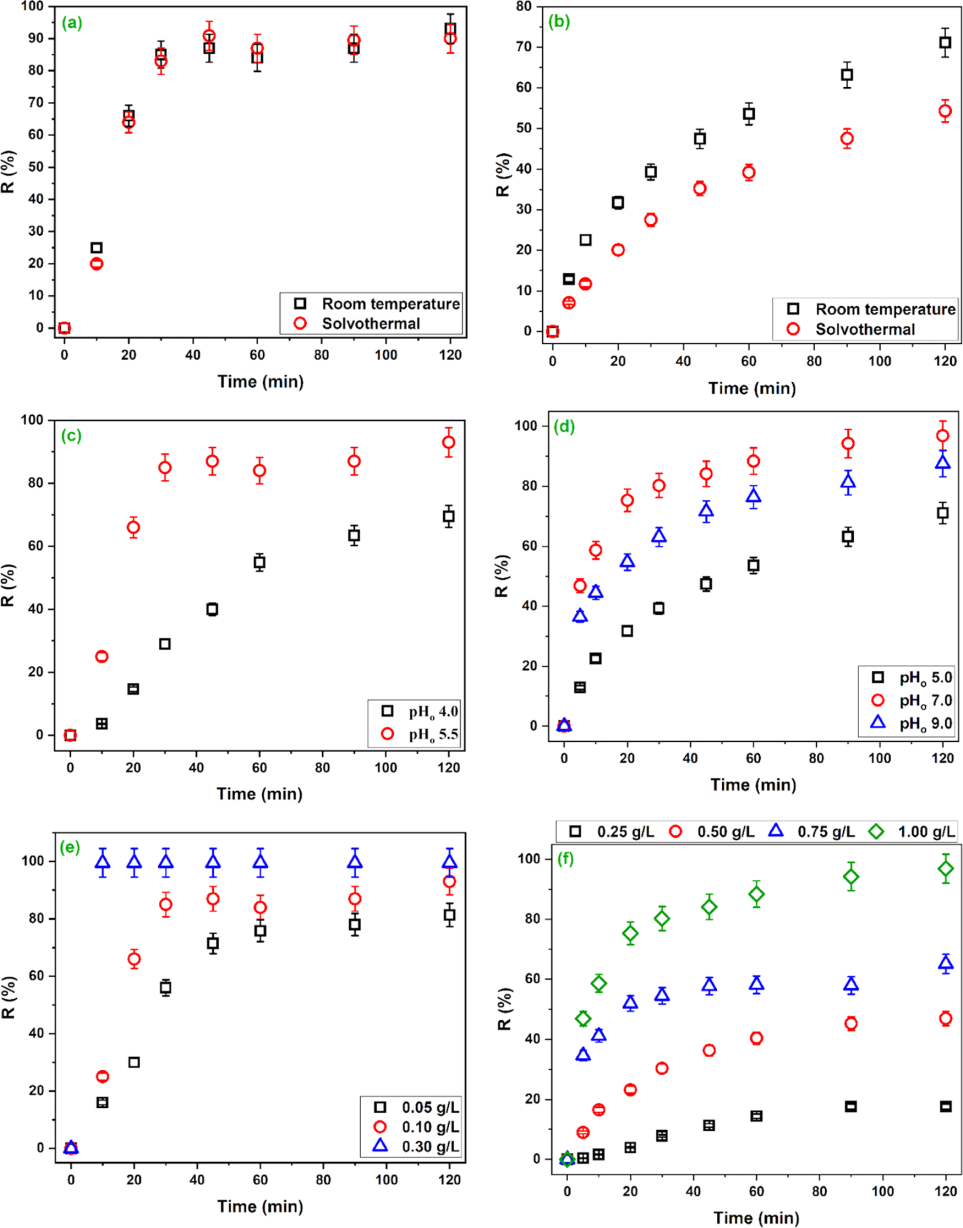
Comparing the efficiency of the FeCo alloy prepared at room temperature and the solvothermal method for the removal of (a) Cu^2+^ (*C_i_* 10 mg/L, pH_o_ 5.5, dosage 0.10 g/L) and (b) RB5 (*C_i_* 10 mg/L, pH_o_ 5, dosage 1.00 g/L). Change of *R* % of (c) Cu^2+^ (*C_i_* 10 mg/L, dosage 0.10 g/L) and (d) RB5 (*C_i_* 10 mg/L, dosage 1.00 g/L) at different pH_o_ and (e) Cu^2+^ (*C_i_* 10 mg/L, pH_o_ 5.5) and (f) RB5 (*C_i_* 10 mg/L, pH_o_ 7) by different dosages of the FeCo alloy prepared at room temperature.

The performance of both FeCo samples for the removal of Cu^2+^ and RB5 was evaluated for comparison purposes, and the results are graphed in Figure 4. Around 90% of Cu^2+^ was removed by both samples after 45 min of contact time (Figure 4a). Thus, it can be concluded that both samples have identical performance for the removal of Cu^2+^. These results are logical and in line with the conclusion that the characteristics of both materials are almost identical based on the discussion in the characteristics of materials section.

On the other hand, after 120 min of contact time, the sample prepared at room temperature removes 71% of the RB5, while the sample prepared by the solvothermal method removes only 54% (Figure 4b). These results look odd since the characteristics of both materials are very similar, as illustrated above. However, a probable reason for the observed results can be deduced from the SEM and TEM results. The results of SEM and TEM proved that the sample prepared by the solvothermal method is more aggregated than the sample prepared at room temperature. The aggregation blocks some adsorption sites and increases the diffusion path length, which result in decreasing the removal.^39,40^ Thus, RB5 removal is lower by the sample prepared by the solvothermal method.

The difference in the behavior of RB5 and Cu^2+^ can be attributed to the difference in their sizes and removal mechanism. The RB5 dye has a molecular size of 29.9 × 8.75 Å,^41^ while Cu^2+^ has a hydrated atomic radius of 4.19 Å.^42^ Thus, contrary to RB5, aggregation has no effect on Cu^2+^ removal as it has a small atomic radius. Based on these results, the sample prepared at room temperature was selected for further investigation.

The solution’s initial pH affects the speciation and form of the adsorptive and the composition of the adsorbent. Copper precipitates as Cu(OH)_2_ starting from pH 6.0;^43^ thus, we limited the study of the pH effect to pH_o_ 5.5. On the other hand, acidic pH accelerates the dissolution of the zero-valent bimetallic nanoparticles,^44^ causing an undesirable increase in the concentration of the metals in the aqueous solution. Therefore, we limited the study of pH effect to pH_o_ 4.

**Figure 5:**
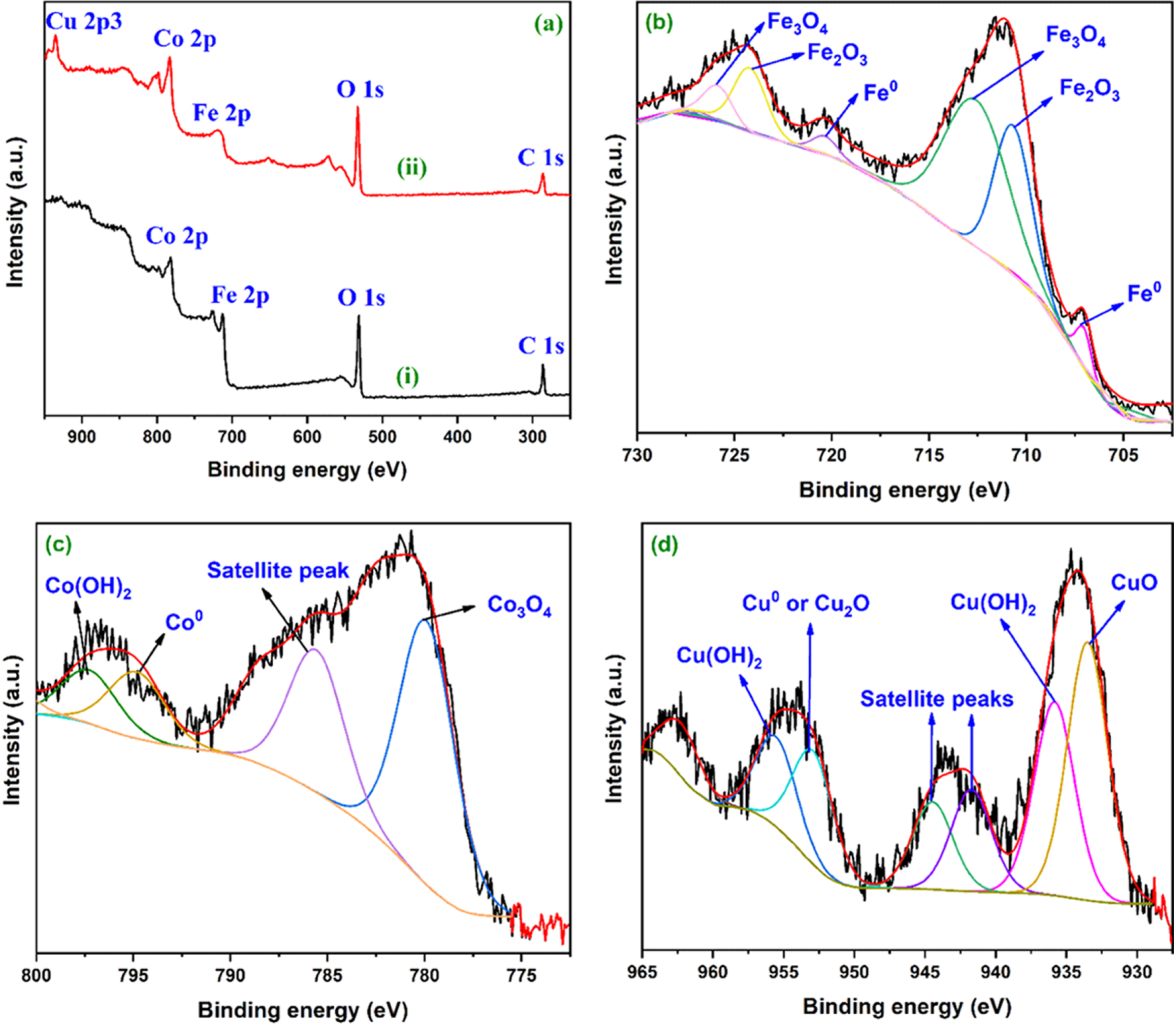
XPS analysis of bimetallic zero-valent FeCo nanoparticles prepared by the room-temperature method. (a) Survey scan before (spectrum i) and after (spectrum ii) contact with Cu^2+^ solution (10 mg/L, pH_o_ 5.5), and narrow-scan spectra of (b) Fe 2p, (c) Co 2p, and (d) Cu 2p.

Figure 4c shows that the Cu^2+^ removal increased from 70 to 93% by increasing the pH_o_ from 4 to 5.5. As illustrated above, the surface of FeCo is positively charged up to pH 8.3. Therefore, repulsion between Cu^2+^ and FeCo dominates under experimental conditions. Thus, the probability of Cu^2+^ adsorption onto FeCo via electrostatic forces can be precluded. It is well known that Fe^0^ is a powerful reductant in water. The standard reduction potential of Cu^2+^ is much more positive than Fe^0^, so it can rapidly reduce to Cu^0^ and/or Cu_2_O and the reduction process is thermodynamically preferred over precipitation and sorption processes.^45,46^ Thus, herein, Cu^2+^ reduction is the most probable removal mechanism. To verify this assumption, XPS analysis was performed before and after contact of the FeCo alloy with a Cu^2+^ solution. Figure 5 shows the obtained XPS survey and narrow-scan spectra. The survey scan (Figure 5a) revealed that the curve shape and peak position of the two samples are very similar. Also, the survey scan exposed the presence of Fe, Co, O, and C elements in both samples and the appearance of a new Cu peak in the sample exposed to the Cu^2+^ solution. The appearance of a Cu peak demonstrates the uptake of Cu by the FeCo alloy. The presence of C might be due to the usage of carbon as the standard XPS correction, while the presence of O indicated the surface oxidation of both samples. The emergence of O in the fresh FeCo alloy does not contradict the above-discussed XRD and EDX results, which revealed the absence of oxides because XPS gives information about the surface elements with a photoelectron probing depth of a few angstroms.^33^ Similar trends of XRD, EDX, and XPS have been reported before.^33,34,47,48^ Based on the results of XRD, EDX, and XPS, it can be concluded that a thin passive oxide layer is formed over the FeCo nanoparticles. This layer protects the FeCo alloy against further air oxidation.^49,50^ The narrow-scan analysis of Fe 2p (Figure 5b) disclosed the presence of Fe^0^ (peaks at around 706 and 719 eV), Fe_2_O_3_ (peaks at 710 and 724 eV), and Fe_3_O_4_ (peaks at 712 and 725 eV). On the other hand, the narrow-scan spectrum of Co 2p (Figure 5c) revealed the presence of peaks at Co^0^ (peak at 793 eV), Co_3_O_4_ (peak at 780 eV), and Co(OH)_2_ (peak at 796 eV). The peak at 785 eV is the satellite peak of the Co 2p_3/2_ main line.^33,34,47,48^

Finally, the narrow-scan spectrum of Cu 2p (Figure 5c) showed a peak at 933.5 eV corresponding to CuO;^51,52^ doublet peaks at 935.8 and 955.5 eV, which are characteristic for Cu(OH)_2;_^51,53^ satellite peaks at 940, 943, and 962 eV; and a peak at 952.9, which could be due to Cu^0^ or Cu_2_O.^51,54^ The differentiation between Cu^0^ and Cu_2_O from the Cu 2p XPS spectra is difficult because they have identical binding energies (±0.1 eV).^52,55^ However, the difference in binding energy between the Cu 2p_3/2_ and Cu 2p_1/2_ peaks is about 20 eV, which supports the presence of Cu^0^ or Cu^+^ on the FeCo alloy surface.^52^

The presence of Cu^0^ or Cu_2_O on the surface of the FeCo alloy corroborates the reduction of Cu^2+^ by the FeCo alloy. Similar results have been reported frequently for the removal of Cu^2+^ by Fe^0.^^45,46^ The appearance of CuO and Cu(OH)_2_ on the surface of FeCo is likely due to the reoxidation of the deposited Cu^0^ or Cu_2_O. The ease of reoxidation of Cu^0^ deposited on Fe^0^ to CuO and Cu(OH)_2(s)_ has been reported previously.^46,56^ Therefore, the XPS analysis supports the assumption that Cu^2+^ removal by the FeCo alloy occurs via the reduction mechanism.

In aqueous solutions, a passive layer of oxides is formed on the surface of the FeCo alloy, as illustrated by XPS analysis (Figure 5). Acidic pH dissolves this passive layer and exposes the reactive sites and therefore is expected to improve the reduction efficiency of the bimetallic nanoparticles. Thus, the removal of Cu^2+^ is expected to be higher at pH_o_ 4. However, this is not the observed case herein: the Cu^2+^ removal was higher at pH_o_ 5.5. A probable reason is that at pH_o_ 4 a proportion of the removed Cu^2+^ undergoes dissolution again, causing an overall decrease in Cu^2+^ removal. A similar effect of pH on copper reduction by Fe^0^ has been observed by Crane et al.^57^

The effect of pH_o_ on RB5 removal displayed in Figure 4d indicates that FeCo can remove RB5 at the different studied pH_o_. However, the highest removal was observed at pH_o_ 7. RB5 is an anionic reactive dye of the vinyl sulfone type that contains an amino, a hydroxyl, and four sulfonate groups (see Figure S1). The sulfonate groups have a very low pKa that can reach a negative value,^58^ so they remain in the anionic form under the experimental conditions. The RB5 dye has pKa at 3.9 (corresponding to the −NH_2_ group) and 6.9 (corresponding to the −OH group).^58^ Thus, at pH_o_ 5, the surface of FeCo is positively charged, and the hydroxyl group of the RB5 dye becomes protonated, while the sulfonate groups are negatively charged and the amino group has its lone pair of electrons free. Thus, attraction between the opposite charges drives the removal process.^59^ At pH_o_ 7, the lone pair of electrons of the hydroxyl groups participates in the interactions with the positively charged surface of FeCo, which results in increasing the removal percentage. At pH_o_ 9, the surface of FeCo is negatively charged and repulsion with the anionic RB5 occurs, which results in a lower removal.^59^ Noteworthy, the observed removal at pH_o_ 9 suggests that electrostatic interactions are not the sole mechanism for the removal of RB5. According to the obtained results, pH_o_ values of 5.5 and 7.0 were used for Cu^2+^ and RB5, respectively, in further studies.

Figure 4e,f shows the change in the percentages of Cu^2+^ and RB5, respectively, removed by different amounts of FeCo prepared at room temperature. The typical trend of increasing the removal percentage with increasing the dosage of a material can be observed for both adsorptives. This trend is an axiomatical result of increasing the number of active sites on increasing the amount of material. Figure 4e shows that Cu^2+^ removal percentages after 120 min. were 81.3, 93.0, and 99.5% for 0.05, 0.10, and 0.30 g/L, respectively. Therefore, a considerable increase in the removal was achieved by increasing the dosage from 0.05 to 0.10 g/L, but a further increase to 0.30 g/L had an insignificant effect on Cu^2+^ removal likely due to approaching complete removal.^39^ On the other hand, an invariably significant increase in the removal of RB5 can be observed by increasing the dosage over the whole studied contact time (Figure 4f). After 120 min., the removal percentages were 17.6, 47.0, 65.1, and 96.9% for 0.25, 0.50, 0.75, and 1.00 g/L, respectively. Therefore, further studies were conducted using dosages of 0.10 g/L for Cu^2+^ and 1.00 g/L for RB5.

The kinetic of removal of Cu^2+^ and RB5 was investigated to get insights into the removal mechanism. Fitting of three kinetic models, pseudo-first order (PFO), pseudo-second order (PSO), and Elovich, to the experimental kinetic data was evaluated. Figure S2 displays the experimental kinetic data and the fitted models. The calculated kinetic parameters and error values are presented in Table 3. The PFO can describe the removal of Cu^2+^ better than the other investigated models as it has a higher correlation coefficient (*R*^2^) and a lower chi-square (χ^2^) and root-mean-square error (RMSE). In addition, the value of *q*_e_ calculated from the PFO was closer to the experimental value. This result matches the previous reports that Cu^2+^ reduction by Fe^0^ follows the PFO kinetic.^60,61^

**Table 3:** Calculated Kinetic Parameters and Error Functions

		Cu^2+^	RB5
experimental *q*_e_ (mg/g)		93.00	3.28
PFO	*R*^2^	0.955	0.969
	χ^2^	62.86	0.038
	RMSE	7.93	0.196
	*k*	0.06 ± 0.01	0.11 ± 0.02
	*q*_e_	91.83 ± 4.90	3.02 ± 0.09
PSO	*R*^2^	0.928	0.995
	χ^2^	99.59	0.006
	RMSE	9.98	0.077
	*k*	5.78 × 10^4^ ± 2.76 × 10^4^	0.05 ± 0.00
	*q*_e_	109.13 ± 11.27	3.36 ± 0.05
Elovich	*R*^2^	0.892	0.994
	χ^2^	149.60	0.007
	RMSE	12.23	0.083
	α	13.40 ± 10.54	2.31 ± 0.55
	β	0.04 ± 0.01	1.85 ± 0.11

On the other hand, the experimental kinetic data of RB5 was best described by the PSO model because it demonstrated the highest *R*^2^ and lowest χ^2^ and RMSE values among the tested models. The close match between the *q*_e_ calculated from the PSO and the experimental one further proved the best fitting of the PSO to the experimental data. The PSO assumes that (i) the rate of adsorption depends on both the adsorptive and the adsorbent; (ii) the adsorption step, not the mass transfer, is the rate-determining step; and (iii) adsorption is due to physiochemical interactions between the adsorptive and adsorbent.^62^ Noteworthy, the Elovich model also gives a good fit to the kinetic data. Typically, the Elovich model is applicable to chemisorption onto a heterogeneous surface.^63^ Thus, the results of RB5 adsorption kinetic modeling suggest the chemisorption process and heterogenous surface of FeCo. This suggestion will further be confirmed by the isotherm study.

**Figure 6:**
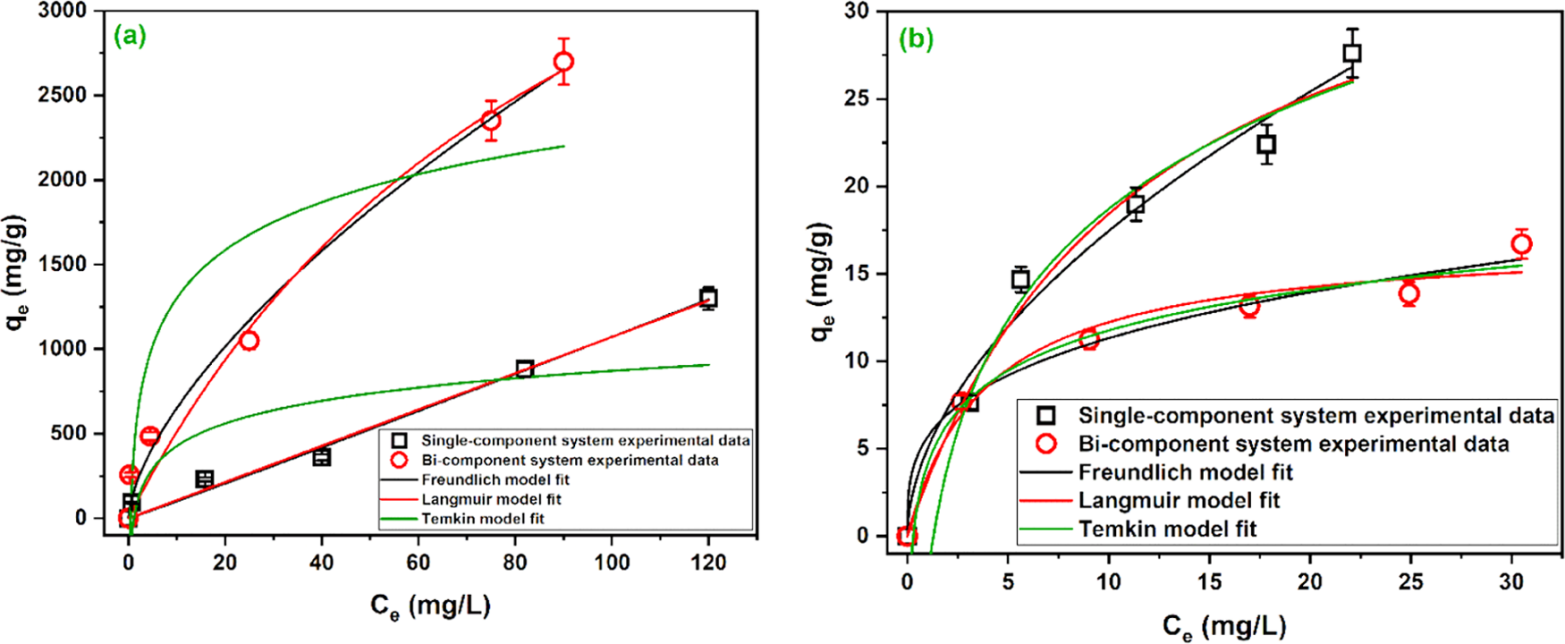
Adsorption isotherm and fitted models for the adsorption of (a) Cu^2+^ and (b) RB5 onto the FeCo alloy prepared at room temperature in single- and bicomponent systems. Single-component system Cu^2+^ (pH_o_ 5, dosage 0.10 g/L) and RB5 (pH_o_ 5, dosage 1.00 g/L). Bicomponent system pH_o_ 5, dosage 1.00 g/L.

The practical isotherm data and the nonlinear fit of different isotherm models are given in Figure 6. The shape of the practical isotherm of Cu^2+^ in single- and bicomponent systems (Figure 6a) can be classified as C1 and L1 curves of the Giles classification, respectively,^64^ while the adsorption isotherm of RB5 in both single- and bicomponent systems (Figure 6b) can be classified as L1. The C1 curve indicates (i) probable penetration of Cu^2+^ into the FeCo alloy and (ii) the creation of more reactive sites as long as Cu^2+^ is removed. On the other hand, the L1 curve indicates that as more adsorptive is adsorbed, finding a vacant adsorption site becomes difficult.^64^

Comparing the adsorption isotherms of Cu^2+^ in the single- and bicomponent systems (Figure 6a) reveals that Cu^2+^ removal in the bicomponent system is much higher than in the single-component system. It seems that the adsorbed RB5 adds negatively charged functional groups on the surface of FeCo, which enhances the adsorption of Cu^2+^ through electrostatic interactions. As illustrated above, at pH_o_ 5, RB5 is adsorbed onto FeCo through the electrostatic interactions between the positively charged surface of FeCo from one side and negatively charged sulfonate groups and electron-rich amino groups from the other side. The unbonded negatively charged and electron-rich functional groups of the RB5 adsorbed on the surface of FeCo act as adsorption sites for Cu^2+^. This synergistic effect is known as “anion-synergism” and has been reported frequently for the simultaneous adsorption of anionic dyes and cationic trace metals.^65−68^

Inversely, comparing the adsorption isotherms of the single- and bicomponent systems of the RB5 dye (Figure 6b) reveals that the FeCo alloy can remove 10 mg/L RB5 in both systems with the same efficiency. However, when the initial concentration of RB5 increases, the removal in the bicomponent system becomes lower than those in the single-component system. This trend might be ascribed to (i) exhaustion of some active sites of FeCo by Cu^2+^ and/or (ii) altering the adsorption characteristics of FeCo as a consequence of the reduction and probable diffusion of Cu^2+^ into the FeCo alloy. The latter reason can be verified from the isotherm models as below.

The parameters of the tested isotherm models are given in Table 4. The analysis of *R*^2^ indicates that the Cu^2+^ removal in single- and bicomponent systems can be described by both Freundlich and Langmuir models. The values of error functions decisively indicate that Freundlich is the best model that fit the isotherm data of Cu^2+^. Additionally, the calculated parameters of the Langmuir model in the single-component system look unrealistic. The value of the Freundlich parameter *n* in the single-component system was almost 1, indicating a linear process. However, in the bicomponent system, the value of *n* was increased to 1.56, indicating the favorability of the adsorption process and increasing the heterogeneity of the FeCo surface.

**Table 4:** Calculated Isotherm Parameters and Error Functions

		Cu^2+^		RB5	
		single-component	bicomponent	single-component	bicomponent
Freundlich	R^2^	0.99	0.99	0.98	0.99
	χ^2^	3923	15201	2.27	0.47
	RMSE	62.63	123.29	1.51	0.69
	*k*_F_	9.52 ± 4.87	14.21 ± 44.31	5.00 ± 0.91	5.67 ± 0.61
	*N*	0.97 ± 0.11	1.56 ± 0.17	1.84 ± 0.23	3.33 ± 0.40
Langmuir	*R*^2^	0.99	0.98	0.98	0.97
	χ^2^	3975	31703	2.27	1.17
	RMSE	63.05	178.05	1.51	1.08
	*k*_L_	1.82 × 10^–6^ ± 0.00	0.01 ± 0.006	0.09 ±0.03	0.26 ± 0.08
	*q*_L_	5.89 × 10^6^ ± 4.83 × 10^9^	5563 ± 1894	39.72 ± 5.22	17.02 ± 1.25
Temkin	*R*^2^	0.70	0.83	0.98	0.99
	χ^2^	95634	271065	1.92	0.65
	RMSE	309.25	520.64	1.39	0.81
	*b*_T_	12.87 ± 5.03	6.08 ± 1.64	272.01 ± 25.42	748.03 ± 93.42
	*A*_T_	0.91 ± 1.24	2.41 ± 2.56	0.77 ± 0.15	3.44 ± 1.66

On the other hand, according to the values of *R*^2^, the Freundlich, Langmuir, and Temkin models can describe the adsorption isotherm of RB5 in single- and bicomponent systems. Once again, the values of error functions show that the Temkin model is the best model that fits the isotherm data of RB5 in the single-component system. However, in the bicomponent system, a better fit can be observed in the case of the Freundlich model. Both Freundlich and Temkin models are nonideal models that suppose a multilayer adsorption. They differ in their assumptions of (i) the relation between the heat of adsorption and surface coverage: Freundlich assumes a logarithmic relationship, while Temkin assumes a linear relationship; and (ii) the binding energy of adsorption sites: Freundlich assumes energetically nonuniform adsorption sites, while Temkin assumes uniform adsorption sites.^69,70^

A comparison of the value of Langmuir q_m_ of the single- and bicomponent systems (Table 4) indicates that the coexistence of RB5 and Cu^2+^ in solution decreased the adsorption of RB5. The values of the Freundlich parameter *n* are greater than 1 in both single- and bicomponent systems, indicating a favorable adsorption process. In addition, the value of the Freundlich heterogeneity factor (*n*) in the bicomponent system is significantly higher than in the single-component system, suggesting an increase in the surface heterogeneity likely due to the reduction and probable diffusion of Cu^2+^ into the FeCo alloy. Thus, the isotherm study elucidated that (i) the adsorption of RB5 and Cu^2+^ onto FeCo is a complicated process that has a multilayer character, (ii) FeCo can simultaneously remove RB5 and Cu^2+^ with more selectivity toward Cu^2+^, and (iii) the simultaneous adsorption of RB5 and Cu^2+^ slightly changes the surface characteristics of the FeCo alloy. In addition, this change was, obviously, associated with a considerable decrease in the adsorption efficiency of FeCo toward RB5.

Usually, the Langmuir monolayer saturation capacity is used to compare the adsorption efficiency of different materials toward a specific contaminant.^71^ Unfortunately, the value of *q*_L_ in the case of Cu^2+^ looks unrealistic, so it cannot be reliably used for comparison with other adsorbents. On the other hand, as the Langmuir model can describe the adsorption isotherm of RB5 onto the FeCo alloy well, the value of *q*_L_ can be used for such a comparison. Table 5 lists the values of *q*_L_ of different reported adsorbents along with that of the FeCo alloy prepared in this work toward the RB5 dye in a single-component system.

**Table 5:** Values of Langmuir Monolayer Adsorption Capacity of Different Adsorbents toward the RB5 Dye

adsorbent	*q*_L_ (mg/g)
peanut hull^72^	50.00
coral-like hierarchical magnesium oxide-incorporated fly ash composite^73^	48.78
FeCo alloy (this work)	39.72
dolomite treated at 900 °C^74^	38.46
carob waste-derived nanoporous carbon^75^	36.90
Na-X zeolite^76^	25.3
magnetite-impregnated activated carbon (NH_4_OH precipitation)^77^	21.91
coconut-shell-based microporous carbon^77^	17.35
textile sludge-activated carbon^78^	11.98
ferrite bismuth nanoparticles^79^	9.65
magnetite-impregnated activated carbon (NaOH precipitation)^77^	2.26
*Eichhornia crassipes*/chitosan	0.606

It can be seen from Table 5 that the prepared FeCo alloy has a higher *q*_L_ than several reported adsorbents. However, there are still some materials that have higher *q*_L_ values. For example, Tanyildizi^72^ and Kumar et al.^73^ have reported a higher *q*_L_ for RB5 adsorption onto peanut hull and the coral-like hierarchical magnesium oxide-incorporated fly ash composite, respectively. However, the FeCo alloy is more practicable than these adsorbents owing to its ease of separation by applying a magnetic field after the treatment of contaminated water. Overall, the comparison in Table 5 shows that the prepared FeCo alloy is a good choice for practical application for the removal of RB5 and similar anionic dyes from contaminated water.

## 4 Conclusions

A simple and quick preparation method for FeCo preparation based on hydrazine reduction at room temperature was revisited with the aim of evaluating the effect of initial salt composition on the purity and particle size of the product and comparing it with the solvothermal method. The results indicated the dependency of the purity and particle size of the produced FeCo on the initial salt composition used. Using nitrate salts or chloride salts of both of iron and cobalt produces a pure crystalline bimetallic zero-valent FeCo alloy. The particle size of FeCo prepared using chloride salts was larger than that prepared using nitrate salts. Solvothermal treatment of the sample prepared using chloride salts reduces the particle size to be comparable to that prepared using nitrate salts at room temperature. Furthermore, the characteristics of FeCo prepared by solvothermal and room-temperature methods using nitrate salts were almost the same. Thus, it can be concluded that room-temperature reduction of iron and cobalt nitrate salts is more advantageous than the solvothermal method. The advantages of the room-temperature method include its fast speed, simplicity, minimal energy consumption, and not requiring protection against oxidation by an inert gas or any agent.

Studies on the efficiency of the prepared FeCo for the removal of Cu^2+^ and RB5 dye in the single-component system illustrated that 0.3 g/L FeCo can completely remove 10 mg/L Cu^2+^ at pH_o_ 5.5 in 10 min. However, the complete removal of 10 mg/L RB5 requires 90 min of contact with 1.0 g/L FeCo at pH_o_ 7. The pseudo-first-order and pseudo-second-order models were best suited to the removal kinetics of Cu^2+^ and RB5, respectively. The FeCo can simultaneously remove Cu^2+^ and RB5 dye. However, a considerable decrease in the removal of RB5 was observed in the bicomponent system, especially at RB5 concentrations higher than 10 mg/L. Contrarily, a considerable increase in the removal of Cu^2+^ was observed in the bicomponent system. A Langmuir monolayer saturation capacity of 5563 mg/g for Cu^2+^ in the bicomponent system demonstrated the high efficiency of the prepared FeCo for water decontamination.

## Supporting Information Available

Adsorption data analysis, adsorption kinetic models, adsorption isotherm models, error analysis, UV–visible spectrum of the RB5 dye, adsorption kinetics, and fitted models (PDF)
