# Supplementary material for: One-Step Room-Temperature Synthesis of Bimetallic Nanoscale Zero-Valent FeCo by Hydrazine Reduction: Effect of Metal Salts and Application in Contaminated Water Treatment
Source: ACS Omega. 2022 Sep 20;7(39):34810–23. doi: 10.1021/acsomega.2c03128 (PMC9535644; doi:10.1021/acsomega.2c03128)
Supplement: Supplementary file 1 — ao2c03128_si_001.pdf [file ao2c03128_si_001.pdf]

Supporting information

One-step room-temperature synthesis of bimetallic nanoscale zero-valent FeCo by hydrazine reduction: effect of metals salts and application in contaminated water treatment

Asmaa A. Koryam [a], Shaimaa T. El-Wakeel [a*], Emad K Radwan [a], Elham S. Darwish [b], and Azza M. Abdel Fattah [b]

[a] Water Pollution Research Department, National Research Centre, 33 El Buhouth St, Dokki, 12622 Giza, Egypt.

[b] Department of Chemistry, Faculty of Science, University of Cairo, Giza, 12613, Egypt.

Contents

Adsorption data analysis S2

Adsorption kinetic models S2

Adsorption isotherm models S3

Error analysis S3

Figures S5

Figure S1. (a) structure and selected properties, (b) visible spectra of different concentrations, and (c) calibration curve of RB5 dye. S5

Figure S2. Adsorption kinetics and fitted models for the adsorption of (a) Cu^{2+} (C_i 10 mg/L, pH_o 5.3, dosage 0.10 g/L), and (b) RB5 (C_i 10 mg/L, pH_o 7, dosage 1.00 g/L) onto FeCo alloy prepared at room temperature. S6

References S7

[*] Corresponding author: shaimaa_tw@yahoo.com
shaimaa.tw@gmail.com

Adsorption data analysis

The removal performance of the alloy at any time *t* was determined by either the amount of Cu^{2+} and RB5 adsorbed onto one gram of the alloy (q_t, Eq S1) or the removal percentage (R%, Eq. S2).

$$q_t = (C_i - C_t)\frac{\mathrm{V}}{\mathrm{m}} \tag{S1}$$

$$\mathrm{R}\ \% = \left(\frac{C_i - C_e}{C_i}\right)100 \tag{S2}$$

where C_i and C_t (mg/L) are the concentration of Cu^{2+} or RB5 at time 0 and t, respectively, V (L) is the volume of Cu^{2+} or RB5 solution, and m (g) is the used mass of the alloy.

Adsorption kinetic models

Pseudo-first-order model

The rate constant of adsorption is determined from the pseudo-first-order equation given by Lagergren and Svenska [1] as follow:

$$q_t = q_e\ (1 - \mathrm{e}^{-k_1 t}) \tag{S3}$$

where q_e (mg/g) is the adsorption capacity at equilibrium, q_t (mg/g) is the amount of solute adsorbed on the adsorbent at time t, k_1 (min^{-1}). is the pseudo-first order rate constant and t (min) the time.

Pseudo-second-order model

The pseudo-second-order equation [2] based on equilibrium adsorption is expressed as:

$$q_t = \frac{k_2 q_e^2 t}{1 + k_2 q_e t} \tag{S4}$$

where k_2 (g/mg min) is the rate constant of the second-order adsorption.

Elovich model

This model is one of the equations that best describes the activated chemical adsorption. It is suitable in systems that have heterogeneous adsorbing surfaces [3].

$$q_t = \frac{1}{\beta}\ \ln\,(1 + \alpha\beta t) \tag{S5}$$

where q_t (mg/g) is the amount of adsorption at time t = t, β is Elovich constant (g/mg), α is initial adsorption rate (mg/(g min))

Adsorption isotherm models

Freundlich model

This empirical model can be used to describe non-ideal distribution of heat of adsorption and affinities on a heterogeneous surface, it is not restricted to the formation of monolayer [4]. The non-linear form can be presented by Eq. S7.

$$q_e = k_F C_e^{1/n} \quad \text{(S6)}$$

where k_F is Freundlich constant ($mg^{(1-1/n)}L^{(1/n)}/g$) related to adsorption capacity, and n (–) adsorption intensity

Langmuir model

Adsorption isotherm of single adsorptive was analyzed with the Langmuir model, which is used to describe a monolayer adsorption onto the surface of an adsorbent with finite number of identical adsorption sites [5], it can be written in non-linear form as:

$$q_e = \frac{q_L k_L C_e}{1 + k_L C_e} \quad \text{(S7)}$$

where k_L (L/mg) is the Langmuir constant and q_L (mg/g) is the monolayer adsorption capacity of the adsorbent.

Temkin model

Temkin model assumes that adsorption is a multi-layer process, and neglects the extremely low and high concentrations [6]. Eq. S8 gives the non-linear form of this model.

$$q_e = \left(\frac{\mathrm{RT}}{b_T}\right) lnA_T C_e \quad \text{(S8)}$$

where A_T (L/g) is Temkin equilibrium binding constant, b_T (J/mol) is Temkin constants, R is the universal gas constant (8.314 J/mol K), and T is the absolute temperature (K).

Error analysis

The error functions have been designed to evaluate the reliability of the models and identify the best model that describe and foresee the adsorption process [7]. Three error functions were applied

in this study, specifically, coefficient of determination (R^2, Eq. S9), nonlinear chi-square (χ^2, Eq. S10), and root mean square error (RMSE, Eq. S11).

$$R^2 = \frac{\sum \left(q_{e,cal} - \bar{q}_{e,exp}\right)^2}{\sum \left(q_{e,cal} - \bar{q}_{e,exp}\right)^2 - \sum\left(q_{e,cal} - q_{e,exp}\right)^2} \tag{S9}$$

$$\chi^2 = \sum_{i=1}^{N}\left[\frac{\left(q_{e,exp} - q_{e,cal}\right)^2}{q_{e,cal}}\right] \tag{S10}$$

$$RMSE = \sqrt{\frac{1}{N-M}\sum_{i=1}^{N}(q_{e,measured} - q_{e,model})^2} \tag{S11}$$

where $q_{e,exp}$ is the experimental adsorption capacity at equilibrium, $q_{e,cal}$ is the calculated adsorption capacity at equilibrium, N is the number of experimental data, $\bar{q}_{e,exp}$ is the average experimental capacity at equilibrium, and M is the number of variables of the model.

Figures

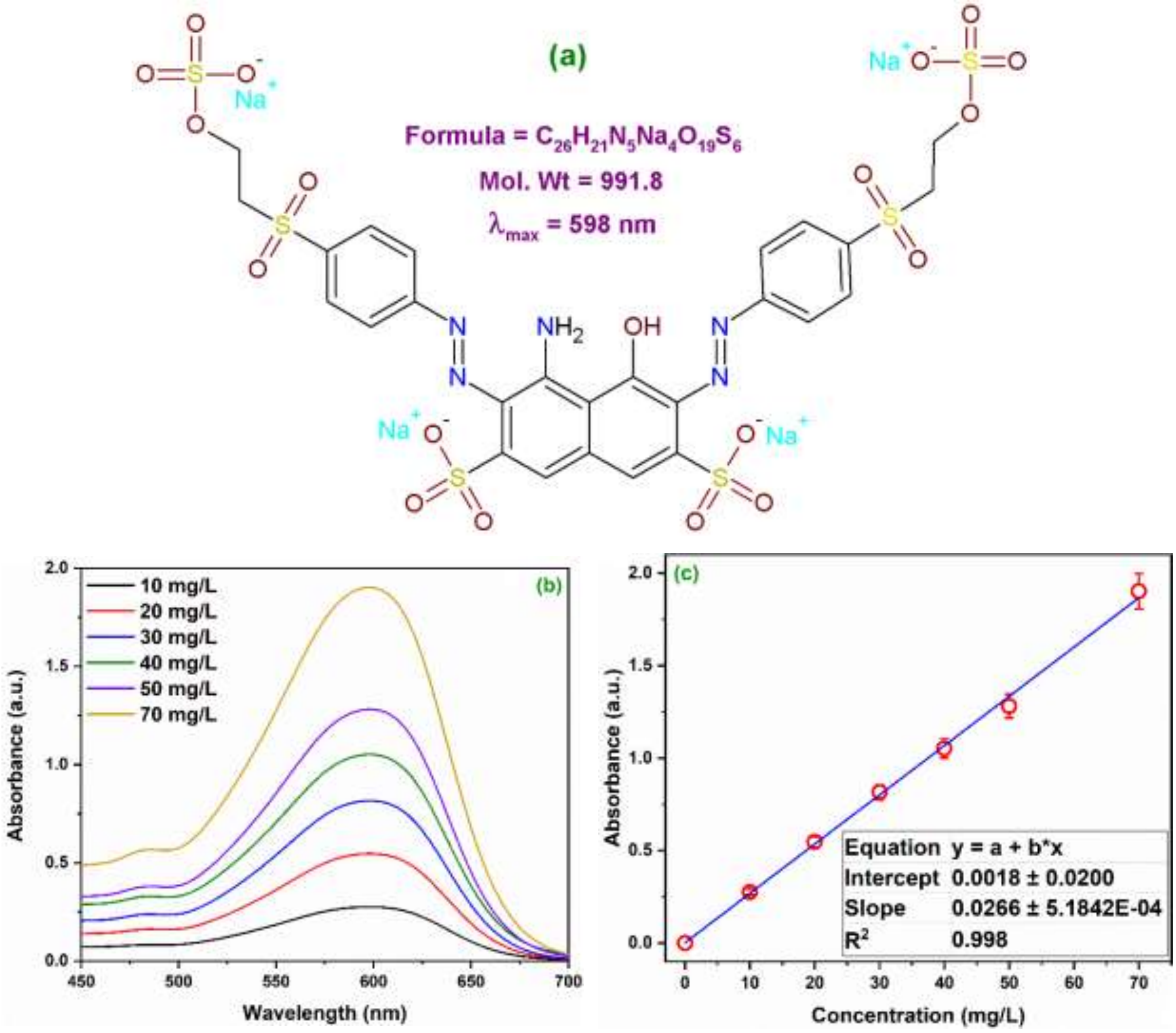

Figure S1. (a) structure and selected properties, (b) visible spectra of different concentrations, and (c) calibration curve of RB5 dye.

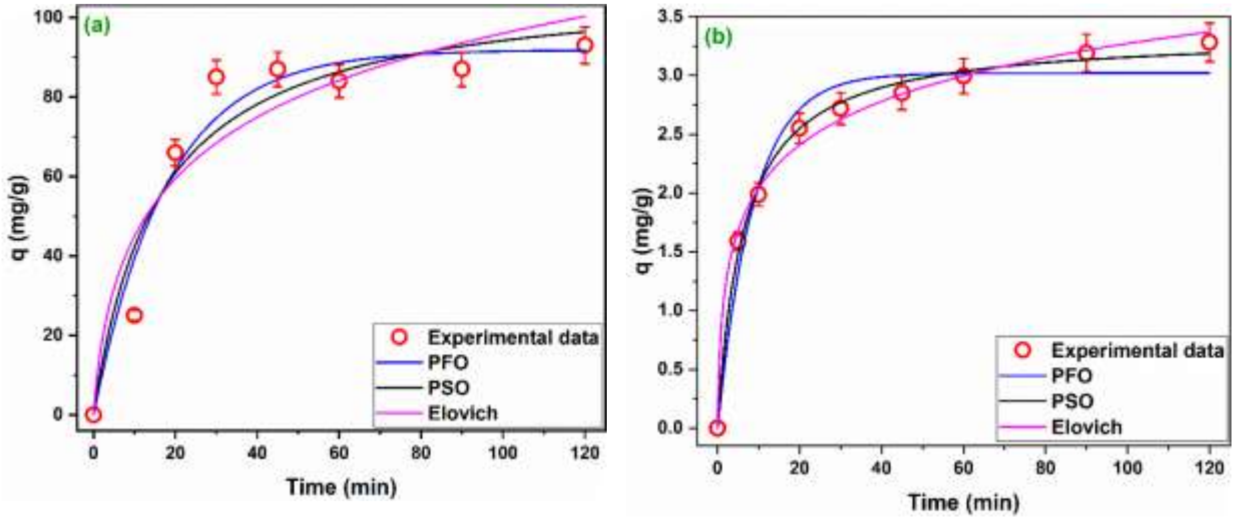

Figure S2. Adsorption kinetics and fitted models for the adsorption of (a) Cu^{2+} (C_i 10 mg/L, pH_o 5.3, dosage 0.10 g/L), and (b) RB5 (C_i 10 mg/L, pH_o 7, dosage 1.00 g/L) onto FeCo alloy prepared at room temperature.

References

(1) Langergren, S.; Svenska, B. K. Zur theorie der sogenannten adsorption geloester stoffe. *Veternskapsakad Handlingar* **1898**, *24* (4), 1-39.

(2) Blanchard, G.; Maunaye, M.; Martin, G. Removal of heavy metals from waters by means of natural zeolites. *Water Res.* **1984**, *18* (12), 1501-1507. DOI: https://doi.org/10.1016/0043-1354(84)90124-6.

(3) Roginsky, S.; Zeldovich, Y. B. The catalytic oxidation of carbon monoxide on manganese dioxide. *Acta Phys. Chem. USSR* **1934**, *1* (554), 2019.

(4) Freundlich, H. M. F. Over the adsorption in solution. *J. Phys. Chem.* **1906**, *57*, 385-470.

(5) Langmuir, I. The adsorption of gases on plane surfaces of glass, mica and platinum. *J. Am. Chem. soc.* **1918**, *40* (9), 1361-1403.

(6) Temkin, M. I.; Pyzhev, V. Kinetics of ammonia synthesis on promoted iron catalyst. *Acta Physiochim.* **1940**, *URSS 12*, 327-356.

(7) Abbas, R.; Kassm, H. An Overview of Using Error function in Adsorption Isotherm Modeling.*MJPS*. **2021**, *8*,In Press.
